# Crystal structure of hexa­glycinium dodeca­iodo­triplumbate

**DOI:** 10.1107/S2056989024007606

**Published:** 2024-08-06

**Authors:** Gayane S. Tonoyan, Gerald Giester, Vahram V. Ghazaryan, Ruben Yu. Chilingaryan, Arthur A. Margaryan, Artak H. Mkrtchyan, Aram M. Petrosyan

**Affiliations:** a Institute of Applied Problems of Physics, NAS of Armenia, 25 Nersessyan Str., 0014 Yerevan, Armenia; b Institute of Mineralogy and Crystallography, University of Vienna, Josef-Holaubek-Platz 2, A-1090 Vienna, Austria; Texas A & M University, USA

**Keywords:** metal–organic compound, iodo­plumbate, crystal structure, tetrel bond, dimeric cation

## Abstract

The crystal structure of hexa­glycinium tetra-μ-iodido-octa­iodido­triplumbate, (C_2_H_6_NO_2_)_6_[Pb_3_I_12_] or (GlyH)_6_[Pb_3_I_12_], is reported. The compound crystallizes in the triclinic space group *P*. The [Pb_3_I_12_]^6−^ anion is discrete and located around a special position: the central Pb ion located on the inversion center is holodirected, while the other two are hemidirected. The supra­molecular nature is mainly based on C—H⋯I, N—H⋯I, O—H⋯I and N—H⋯O hydrogen bonds. Dimeric cations of type (*A*^+^⋯*A*^+^) for the amino acid glycine are observed for the first time.

## Abstract 2

The crystal structure of (GlyH)_6_(Pb_3_I_12_) is reported. Dimeric cations of type (*A*^+^⋯*A*^+^) for the amino acid glycine are observed for the first time.

## 1. Chemical context

Various inorganic and organic–inorganic hybrid materials are used in third-generation photovoltaic devices as solar energy converters (Peng *et al.*, 2015 ▸; Ahmed *et al.*, 2015 ▸; Zhou *et al.*, 2018 ▸).

Haloplumbates were also considered to be ‘solar’ materials (Kojima *et al.*, 2009 ▸; Naza­renko *et al.*, 2018 ▸), but it turned out that plumbates have unfavorable properties, such as instability and toxicity. However, these compounds may have applications in other inter­esting areas: white–light emitting materials (Peng *et al.*, 2018 ▸), luminescent sensing (Wang *et al.*, 2019 ▸; Wang, 2020 ▸; Martínez Casado *et al.*, 2012 ▸), ferroelectric materials (Gao *et al.*, 2017 ▸), non-linear optical materials (Chen *et al.*, 2020 ▸) and semiconductors (Terpstra *et al.*, 1997 ▸).

Our research group has been studying various amino acid salts for a long time (Fleck & Petrosyan, 2014 ▸), and we assumed that amino acids could be used to synthesize organic–inorganic hybrid materials. After the successful synthesis of (GlyH)PbBr_3_ (Tonoyan *et al.*, 2024 ▸), efforts were focused on obtaining (GlyH)PbI_3_.

These compounds are also inter­esting for lead chemistry. Pb^2+^ has an electronic configuration of [Xe]6*s*^2^ 4*f*^14^ 5*d*^10^. The 6*s*^2^ electrons determine the stereochemistry of Pb^II^. Upon hybridization of the *s* and *p* orbitals, the stereochemically active 6*s*^2^ electron pair occupies a position in the coordination sphere of the metal (hemidirected coordination). In this case, such hybridization does not occur, the 6*s*^2^ electron pair has only *s* character and is stereochemically inactive (holodirected coordination) (Casas *et al.*, 2006 ▸; Seth *et al.*, 2018 ▸). As the lead ion has released its two 6*p*^2^ electrons, σ-hole inter­actions are possible. These inter­actions are known among elements of group IV and usually include the tetrel bonding inter­action. In other words, the hemidirectional nature of lead(II) centers is the basic reason for different tetrel bonding inter­actions such as Pb⋯O (S, N, Cl, Br, I), which lead to the formation of supra­molecular assemblies.
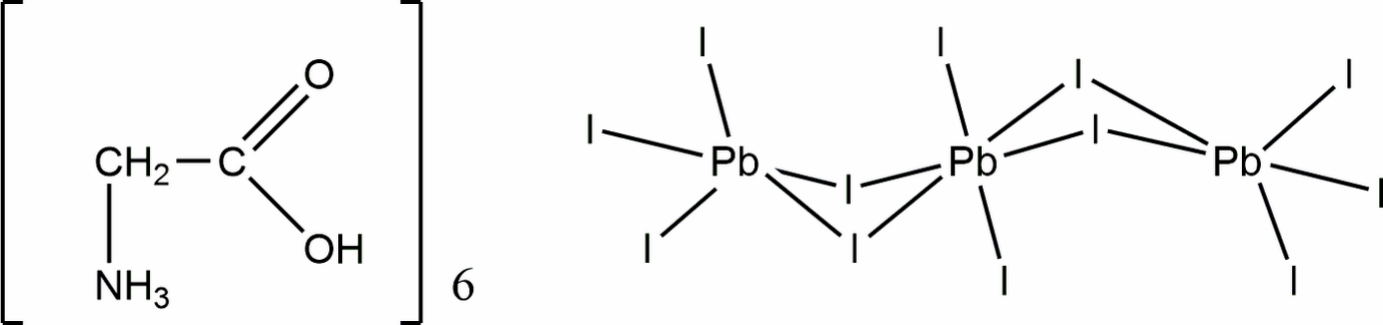


Instead of (GlyH)PbI_3_ crystals, those of (GlyH)_6_(Pb_3_I_12_) were formed unexpectedly. The [Pb_3_I_12_]^6−^ anion is already known (Wang *et al.*, 2015 ▸, 2017 ▸; Lemmerer & Billing, 2012 ▸); it has three lead centers, which can be stereochemically different. In the [Pb_3_I_12_]^6−^ anion of {(tbp)_2_[Pb_3_I_12_]}_*n*_ obtained by Wang *et al.* (2015 ▸), the lead centers are holodirected, coordinated by six iodine atoms, and have an octa­hedral geometry. In (GlyH)_6_(Pb_3_I_12_), the Pb1 center has a holodirected coordination and is bound to six I atoms, while the Pb2 centers with hemidirected coordination are linked to five I atoms. The anion described by Lemmerer & Billing (2012 ▸), as well as that reported by Wang *et al.* (2017 ▸) both correspond to our case considering the long Pb1—I6 distance [3.482 (1) Å]; however, these authors misinter­preted the coordination as holodirected or six-coordinate.

## 2. Structural commentary

**Figure 1:**
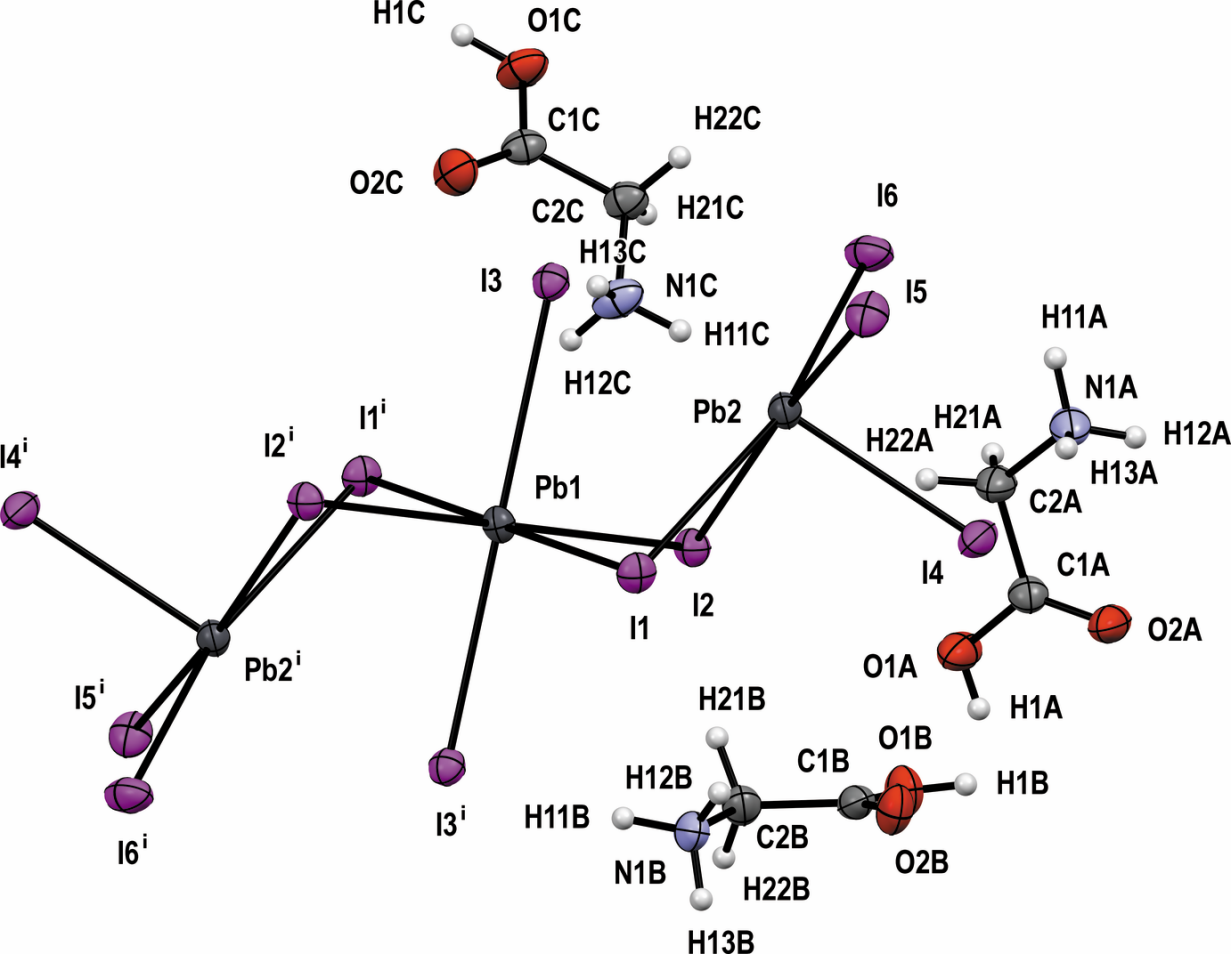
Mol­ecular structure of (GlyH)_6_(Pb_3_I_12_). Symmetry code: (i) −*x* + 1, −*y* + 1, −*z* + 1.

**Figure 2:**
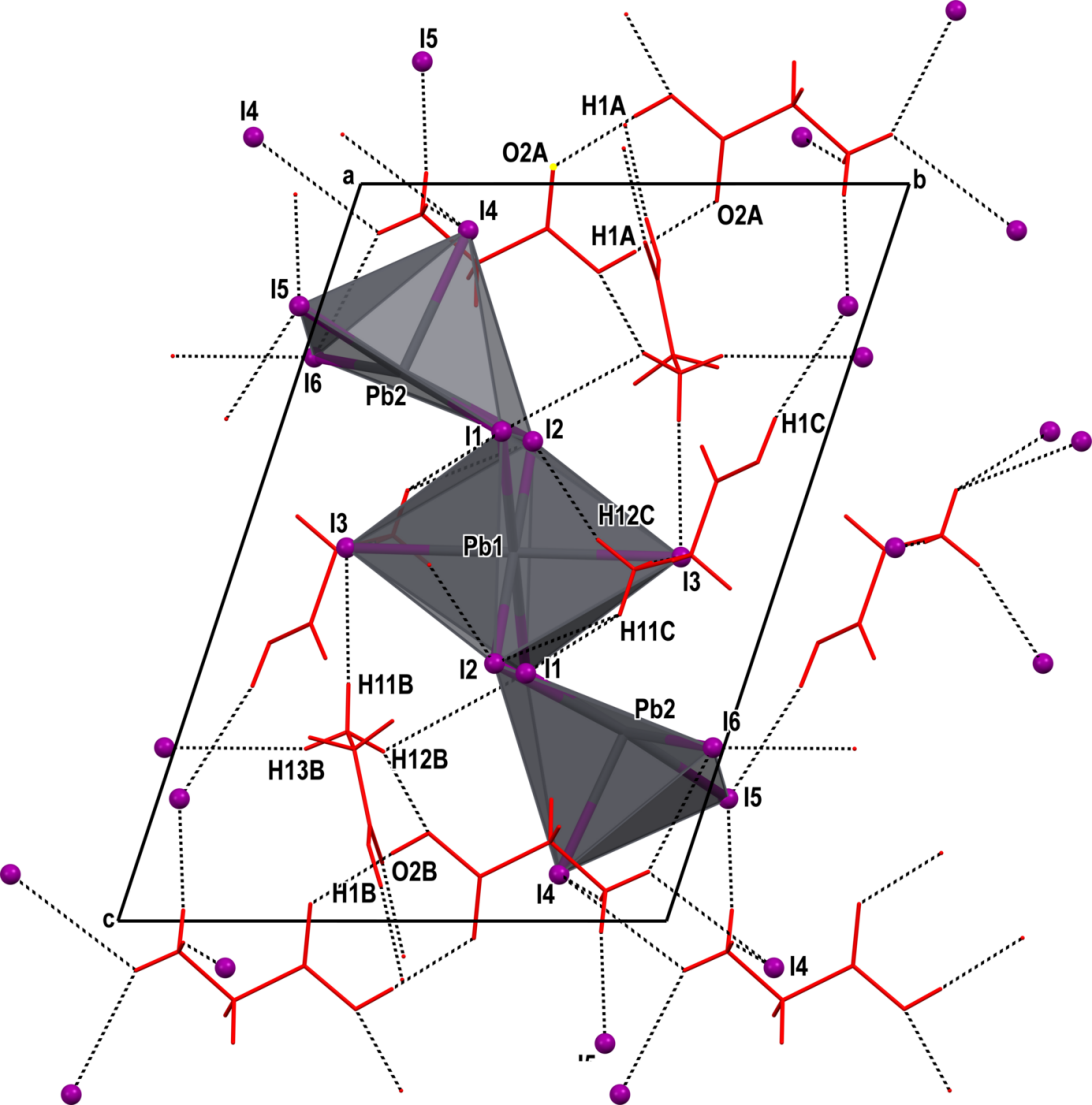
The packing of (GlyH)_6_(Pb_3_I_12_).

**Figure 3:**
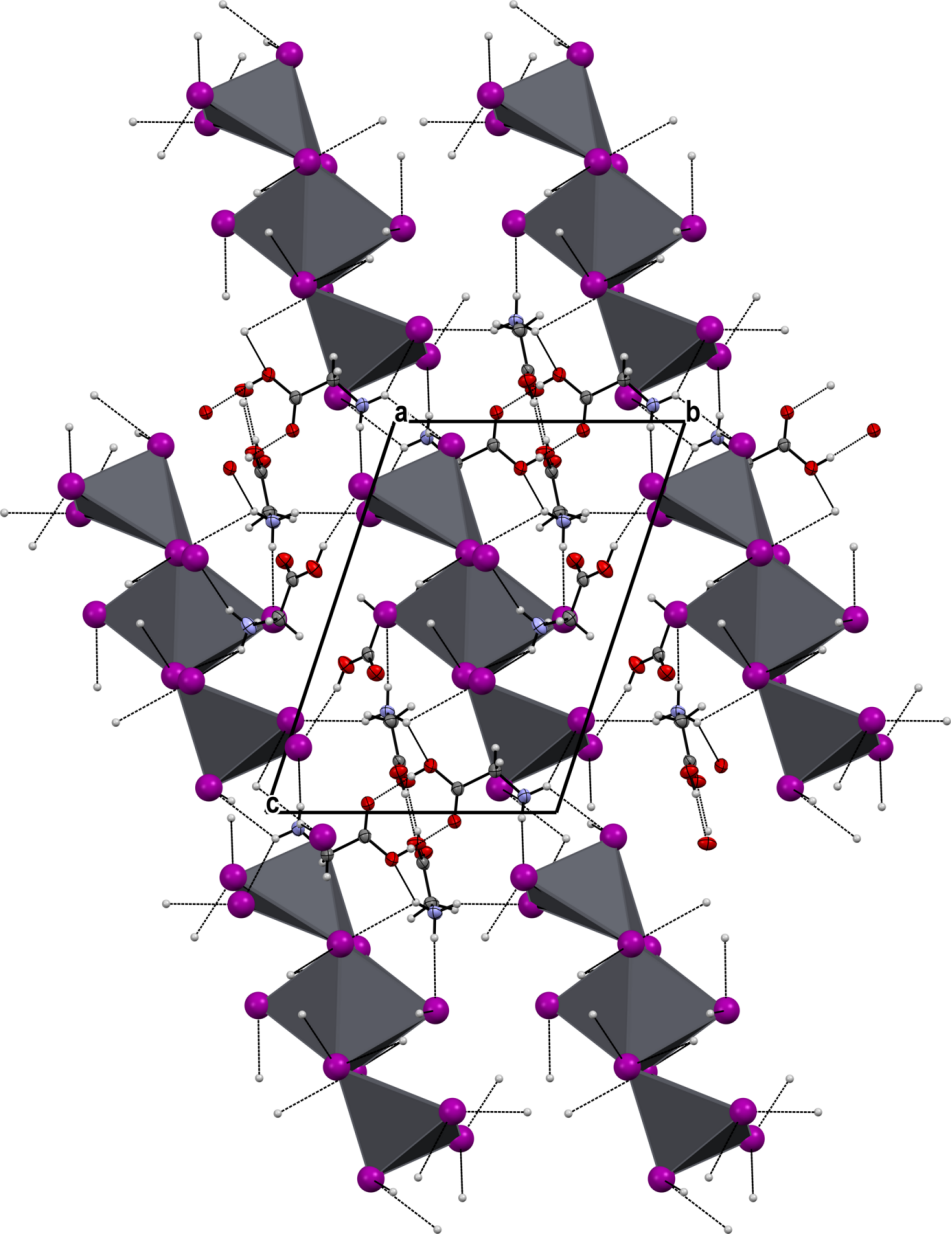
Parallel anions in the packing of (GlyH)_6_(Pb_3_I_12_) viewed along the *a* axis.

The title salt (GlyH)_6_(Pb_3_I_12_) crystallizes in the triclinic space group *P*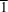 with the asymmetric unit containing half of the formula unit. Selected bond lengths are given in Table 1 ▸ and the mol­ecular structure is shown in Fig. 1 ▸. In (GlyH)_6_(Pb_3_I_12_) the [Pb_3_I_12_]^6−^ anion is discrete. The Pb1 center has a holodirected coordination with six I atoms, thus forming an octa­hedron. The two Pb2 centers have hemidirected coordinations with five I atoms, forming distorted tetra­gonal pyramids. These hemidirected lead ions have stereochemically active lone pairs. Despite this, any donor–acceptor, covalent or tetrel bonds are missing. The lead centers are connected with each other *via* Pb—I—Pb covalent bonds (Fig. 2 ▸). The anions are located parallel to each other, and the glycinium cations cross-link the entire structure through C—H⋯I, N—H⋯I and O—H⋯I hydrogen bonds (Fig. 3 ▸).

## 3. Supra­molecular features

The crystal structure is consolidated *via* O—H⋯O, O—H⋯I, N—H⋯O, N—H⋯I and C—H⋯I hydrogen bonds (Table 2 ▸). The carboxyl group of the glycinium cation *A* forms a hydrogen bond [O1*A—*H1*A*⋯O2*A*, 2.637 (3) Å] with a symmetry-related glycinium *A* cation; the same is the case for the cation *B*: O1*B*—H1*B*⋯O2*B* [2.667 (3) Å]. However, the carboxyl group of the glycinium cation *C* establishes a hydrogen bond O1*C*—H1*C*⋯I5 [3.445 (3) Å] with the anion. Thus, the *A* and *B* glycinium cations form centrosymmetric (*A*^+^⋯*A*^+^) type dimeric cations, which so far have not been reported for glycine (Fleck & Petrosyan, 2014 ▸).

The NH_3_^+^ groups form rather strong: N1*A*—H11*A*⋯I4 [3.549 (3) Å], N1*A*—H12*A*⋯I5 [3.588 (3) Å], N1*B*—H11*B*⋯I3 [3.648 (3) Å], N1*B*—H12*B*⋯I1 [3.647 (3) Å], N1*C*—H13*C*⋯I3 [3.628 (3) Å] and weak: N1*A*—H11*A*⋯I6 [3.655 (3) Å], N1*A*—H13*A*⋯I4 [3.605 (3) Å], N1*B*—H12*B*⋯I1 [3.647 (3) Å], N1*C*—H11*C*⋯I1 [3.711 (3) Å], N1*C*—H11*C*⋯I2 [3.640 (3) Å], N1*C*—H12*C*⋯I2 [3.779 (3) Å] hydrogen bonds with the anions. There are also C—H⋯I-type contacts: C2*A*—H21*A*⋯I1 [3.669 (3) Å], C2*B*—H21*B*⋯I2 [3.976 (3) Å], and C2*C*—H21*C*⋯I3 [4.068 (3) Å], which can be considered as very weak hydrogen bonds. Thus, these glycinium cations cross-link the entire structure and consolidate it.

## 4. Database survey

A survey of the Cambridge Structural Database (CSD2023.2.0, version 5.45, November update; Groom *et al.*, 2016 ▸) revealed several similar structures. Currently, the Cambridge Structural Database contains 23 entries for the [Pb_3_I_12_]^6−^ anion, which can exist in both discrete and polymeric forms that also have different subtypes. In particular, the discrete type has three subtypes: when the middle lead atom of the trinuclear [Pb_3_I_12_]^6−^ anion has one (Leng *et al.*, 2023 ▸), two, or three (Wang *et al.*, 2015 ▸; Yue *et al.*, 2019 ▸; Zhang *et al.*, 2022 ▸) bridging iodine atoms. When there are one or two bridging iodine atoms, the central lead center has a holodirected coordination and the outer lead atoms have a hemidirected coordination. The anions presented in these works (Lemmerer & Billing, 2012 ▸; Wang *et al.*, 2017 ▸; Cheng *et al.*, 2023 ▸) correspond to our case, where the central lead atom has two bridging iodine atoms and the lead centers have different stereochemistry: holodirected (six-coordinate) and hemidirected (five-coordinate). The polymeric [Pb_3_I_12_]^6−^ anion can be linear (Liang *et al.*, 2023 ▸) or cross-linked (Michael & Harald, 2018 ▸; Naza­renko *et al.*, 2018 ▸; Passarelli *et al.*, 2020 ▸). In summary, 15 [Pb_3_I_12_]^6−^ anions from the 23 entries in the CSD are discrete, 7 are polymeric and one case is remarkable (Yao *et al.*, 2022 ▸) with both a polymer and a discrete [Pb_3_I_12_]^6−^ anion being present in the crystal structure.

## 5. Synthesis and crystallization

As initial reagents we used amino acid glycine (99%) and hydriodic acid (57% w/w, distilled, stabilized with <1.5% hypo­phospho­rous acid, 99.95%). Initially, lead and hydriodic acid were taken in a 1:3 stoichiometric ratio. When the amount of acid in the solution decreases, the reaction between metal and acid slows and eventually almost stops (when no H_2_ gas is released). At this point, the amount of obtained lead(II) iodide (PbI_2_) and remaining acid (HI) was calculated (1:6 stoichiometric ratio). Next, the appropriate amount of glycine was added and mixed. The final stoichiometric ratio of Gly, PbI_2_ and HI was 1:1:6. Instead of the desired compound (GlyH)PbI_3_, only (GlyH)_6_(Pb_3_I_12_) was obtained. Light-red, needle-shaped crystals were obtained by solvent evaporation in a closed container, using silica gel as an absorber. (GlyH)_6_(Pb_3_I_12_) is very hygroscopic: in the IR spectrum the absorption band at 3524 cm^−1^ corresponds to the ν(OH) stretching modes of the hygroscopic water mol­ecules. The band with a peak at 3036 cm^−1^ is caused by ν(NH) of the NH_3_^+^ groups of glycinium cations. The peaks at 2916 cm^−1^ and 2854 cm^−1^ are assigned to ν(CH) of the CH_2_ groups, and the strong band at 1716 cm^−1^ to ν(C=O) of the carboxyl groups.

**Figure 4:**
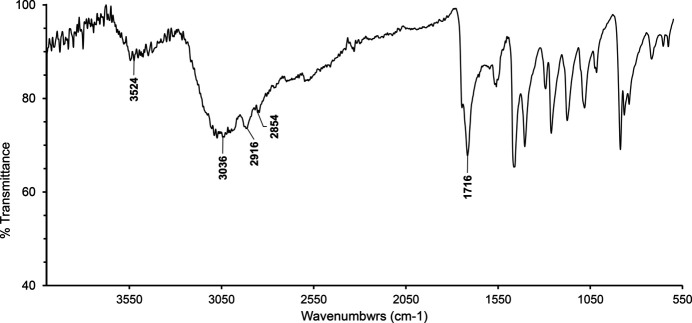
FTIR spectrum of the title compound.

An attenuated total reflection Fourier-transform infrared spectrum (ATR-FTIR) was recorded on an Agilent Cary 630 spectrometer using a germanium (Ge) ATR sampling module (Ge crystal, Happ–Genzel apodization, ATR distortion corrected, 64 scans, 4 cm^−1^ resolution). The IR spectrum is shown in Fig. 4 ▸.

## 6. Refinement

Crystal data, data collection and structure refinement details are summarized in Table 3 ▸. Hydrogen atoms were treated as riding on their parent atoms [C—H = 0.99 Å, N—H = 0.91 Å; *U*_iso_(H) = 1.2U_eq_(C) or *U*_iso_(H) = 1.5U_eq_(N)] except those of the carboxyl group, which were refined with the restraint *U*_iso_(H) = 1.5U_eq_(C).

## Supplementary Material

Crystal structure: contains datablock(s) I. DOI: 10.1107/S2056989024007606/jy2048sup1.cif

Structure factors: contains datablock(s) I. DOI: 10.1107/S2056989024007606/jy2048Isup3.hkl

Supporting information file. DOI: 10.1107/S2056989024007606/jy2048Isup4.mol

Supporting information file. DOI: 10.1107/S2056989024007606/jy2048sup5.docx

CCDC reference: 2368898

Additional supporting information:  crystallographic information; 3D view; checkCIF report

## Figures and Tables

**Table 1:** Selected bond lengths (Å)

C1*A*—O1*A*	1.304 (4)	C1*B*—O1*B*	1.302 (4)	C1*C*—O1*C*	1.322 (4)
C1*A*—O2*A*	1.209 (4)	C1*B*—O2*B*	1.210 (4)	C1*C*—O2*C*	1.196 (4)
C1*A*—C2*A*	1.502 (4)	C1*B*—C2*B*	1.499 (4)	C1*C*—C2*C*	1.507 (5)
C2*A*—N1*A*	1.474 (4)	C2*B*—N1*B*	1.476 (4)	C2*C*—N1*C*	1.477 (4)
					
Pb1—I1	3.1575 (3) 2×	Pb2—I1	3.4049 (3)	Pb2—I4	3.0213 (3)
Pb1—I2	3.1988 (2) 2×	Pb2—I2	3.4063 (3)	Pb2—I5	3.0926 (3)
Pb1—I3	3.2432 (3) 2×			Pb2—I6	3.0663 (3)

**Table 2:** Hydrogen-bond geometry (Å, °)

*D*—H⋯*A*	*D*—H	H⋯*A*	*D*⋯*A*	*D*—H⋯*A*
O1*A*—H1*A*⋯O2*A*^i^	0.79 (5)	1.85 (3)	2.638 (3)	171 (4)
C2*A*—H21*A*⋯I1^ii^	0.99	3.10	3.669 (3)	118
C2*A*—H22*A*⋯I2	0.99	3.33	4.271 (4)	160
N1*A*—H11*A*⋯I4^iii^	0.91	3.05	3.549 (3)	116
N1*A*—H11*A*⋯I5^ii^	0.91	3.27	3.983 (3)	137
N1*A*—H11*A*⋯I6	0.91	3.04	3.655 (3)	126
N1*A*—H12*A*⋯I5^iii^	0.91	2.69	3.588 (3)	170
N1*A*—H13*A*⋯I4	0.91	2.75	3.605 (3)	156
O1*B*—H1*B*⋯O2*B*^iv^	0.82 (4)	1.85 (4)	2.667 (3)	176 (4)
C2*B*—H21*B*⋯I2	0.99	3.03	3.976 (3)	161
C2*B*—H22*B*⋯I6^v^	0.99	3.18	3.775 (3)	120
N1*B*—H11*B*⋯I3^vi^	0.91	2.82	3.648 (3)	151
N1*B*—H12*B*⋯I1	0.91	3.11	3.647 (3)	119
N1*B*—H12*B*⋯O1*A*^vii^	0.91	2.49	3.320 (4)	151
N1*B*—H13*B*⋯I6^v^	0.91	2.79	3.528 (3)	139
O1*C*—H1*C*⋯I5^viii^	0.90 (5)	2.57 (3)	3.445 (3)	164 (4)
N1*C*—H11*C*⋯I1^ii^	0.91	3.09	3.711 (3)	127
N1*C*—H11*C*⋯I2	0.91	3.13	3.640 (3)	117
N1*C*—H11*C*⋯I6	0.91	3.31	3.868 (3)	122
N1*C*—H12*C*⋯I2^ix^	0.91	3.00	3.779 (3)	145
N1*C*—H13*C*⋯I3	0.91	2.73	3.628 (3)	170
C2*C*—H21*C*⋯I3^ii^	0.99	3.14	4.068 (3)	156

Symmetry codes: (i) 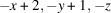; (ii) 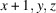; (iii) 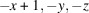; (iv) 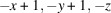; (v) 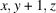; (vi) 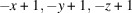; (vii) 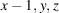; (viii) 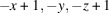; (ix) 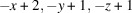.

**Table 3:** Experimental details

Crystal data	
Chemical formula	(C_2_H_6_NO_2_)_6_[Pb_3_I_12_]
*M*_r_	2600.84
Crystal system, space group	Triclinic, *P* 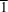
Temperature (K)	200
*a*, *b*, *c* (Å)	8.5437 (5), 11.2672 (7), 14.8534 (9)
α, β, γ (°)	105.900 (2), 92.647 (2), 111.477 (2)
*V* (Å^3^)	1262.40 (13)
*Z*	1
Radiation type	Mo *K*α
μ (mm^−1^)	17.36
Crystal size (mm)	0.1 × 0.08 × 0.06

Data collection	
Diffractometer	Bruker APEXII CCD
Absorption correction	Multi-scan (*SADABS*; Krause *et al.*, 2015 ▸)
*T*_min_, *T*_max_	0.548, 0.746
No. of measured, independent and observed [*I* > 2σ(*I*)] reflections	54402, 9640, 8389
*R*_int_	0.029
(sin θ/λ)_max_ (Å^−1^)	0.771

Refinement	
*R*[*F*^2^ > 2σ(*F*^2^)], *wR*(*F*^2^), *S*	0.022, 0.039, 1.03
No. of reflections	9640
No. of parameters	215
H-atom treatment	H atoms treated by a mixture of independent and constrained refinement
Δρ_max_, Δρ_min_ (e Å^−3^)	2.29, −2.49

Computer programs: *APEX5* Bruker (2024 ▸), *SHELXT2018/2* (Sheldrick, 2015*a* ▸), *SHELXL2019/2* (Sheldrick, 2015*b* ▸), *Mercury* (Macrae *et al.*, 2020 ▸) and *ShelXle* (Hübschle *et al.*, 2011 ▸).

## supplementary crystallographic information

### Hexaglycinium tetra-µ-iodido-octaiodidotriplumbate Crystal data

**Table:**

(C_2_H_6_NO_2_)_6_[Pb_3_I_12_]	*Z* = 1
*M**_r_* = 2600.84	*F*(000) = 1128
Triclinic, *P*1	*D*_x_ = 3.421 Mg m^−^^3^
*a* = 8.5437 (5) Å	Mo *K*α radiation, λ = 0.71073 Å
*b* = 11.2672 (7) Å	Cell parameters from 9918 reflections
*c* = 14.8534 (9) Å	θ = 2.6–32.9°
α = 105.900 (2)°	µ = 17.36 mm^−^^1^
β = 92.647 (2)°	*T* = 200 K
γ = 111.477 (2)°	Block, yellow
*V* = 1262.40 (13) Å^3^	0.1 × 0.08 × 0.06 mm

### Hexaglycinium tetra-µ-iodido-octaiodidotriplumbate Data collection

**Table:**

Bruker APEXII CCD diffractometer	8389 reflections with *I* > 2σ(*I*)
φ and ω scans	*R*_int_ = 0.029
Absorption correction: multi-scan (SADABS; Krause *et al.*, 2015)	θ_max_ = 33.2°, θ_min_ = 2.1°
*T*_min_ = 0.548, *T*_max_ = 0.746	*h* = −13→13
54402 measured reflections	*k* = −17→17
9640 independent reflections	*l* = −22→22

### Hexaglycinium tetra-µ-iodido-octaiodidotriplumbate Refinement

**Table:**

Refinement on *F*^2^	Secondary atom site location: difference Fourier map
Least-squares matrix: full	Hydrogen site location: inferred from neighbouring sites
*R*[*F*^2^ > 2σ(*F*^2^)] = 0.022	H atoms treated by a mixture of independent and constrained refinement
*wR*(*F*^2^) = 0.039	*w* = 1/[σ^2^(*F*_o_^2^) + (0.0087*P*)^2^ + 2.5693*P*] where *P* = (*F*_o_^2^ + 2*F*_c_^2^)/3
*S* = 1.03	(Δ/σ)_max_ = 0.002
9640 reflections	Δρ_max_ = 2.29 e Å^−^^3^
215 parameters	Δρ_min_ = −2.48 e Å^−^^3^
0 restraints	Extinction correction: *SHELXL2019/2* (Sheldrick 2015b), Fc^*^=kFc[1+0.001xFc^2^λ^3^/sin(2θ)]^-1/4^
Primary atom site location: structure-invariant direct methods	Extinction coefficient: 0.00049 (2)

### Hexaglycinium tetra-µ-iodido-octaiodidotriplumbate Special details

**Table:**

Geometry. All esds (except the esd in the dihedral angle between two l.s. planes) are estimated using the full covariance matrix. The cell esds are taken into account individually in the estimation of esds in distances, angles and torsion angles; correlations between esds in cell parameters are only used when they are defined by crystal symmetry. An approximate (isotropic) treatment of cell esds is used for estimating esds involving l.s. planes.

### Hexaglycinium tetra-µ-iodido-octaiodidotriplumbate Fractional atomic coordinates and isotropic or equivalent isotropic displacement parameters (Å^2^)

**Table:**

	*x*	*y*	*z*	*U*_iso_*/*U*_eq_	
Pb1	0.500000	0.500000	0.500000	0.02041 (3)	
Pb2	0.38342 (2)	0.18885 (2)	0.25654 (2)	0.02316 (3)	
I1	0.19064 (2)	0.40522 (2)	0.33606 (2)	0.02537 (4)	
I2	0.74935 (2)	0.46827 (2)	0.34936 (2)	0.02390 (4)	
I3	0.41471 (2)	0.19237 (2)	0.49364 (2)	0.02463 (4)	
I4	0.42551 (3)	0.22381 (2)	0.06337 (2)	0.03591 (5)	
I5	0.03007 (3)	−0.03864 (2)	0.16590 (2)	0.03305 (5)	
I6	0.59810 (3)	0.01929 (2)	0.23522 (2)	0.03749 (6)	
O1A	1.0289 (4)	0.4863 (2)	0.11937 (18)	0.0392 (6)	
H1A	1.044 (5)	0.540 (3)	0.0923 (19)	0.059*	
O2A	0.8915 (4)	0.3408 (2)	−0.02294 (17)	0.0389 (6)	
C1A	0.9400 (4)	0.3667 (3)	0.0605 (2)	0.0263 (6)	
C2A	0.9034 (5)	0.2593 (3)	0.1076 (2)	0.0330 (7)	
H21A	1.011081	0.252552	0.127759	0.040*	
H22A	0.852011	0.282924	0.164719	0.040*	
N1A	0.7854 (4)	0.1293 (3)	0.0409 (2)	0.0366 (7)	
H11A	0.774867	0.062872	0.066806	0.055*	
H12A	0.826984	0.112846	−0.014398	0.055*	
H13A	0.681464	0.132077	0.029277	0.055*	
O1B	0.6686 (3)	0.5891 (3)	0.10354 (18)	0.0352 (6)	
H1B	0.6483 (17)	0.542 (5)	0.048 (3)	0.053*	
O2B	0.3916 (3)	0.5535 (3)	0.07956 (16)	0.0347 (5)	
C1B	0.5289 (4)	0.5982 (3)	0.1296 (2)	0.0270 (6)	
C2B	0.5538 (5)	0.6726 (4)	0.2331 (2)	0.0316 (7)	
H21B	0.578315	0.619973	0.271200	0.038*	
H22B	0.652301	0.759908	0.248893	0.038*	
N1B	0.3990 (4)	0.6954 (3)	0.2566 (2)	0.0316 (6)	
H11B	0.404495	0.721763	0.320722	0.047*	
H12B	0.305401	0.618023	0.229916	0.047*	
H13B	0.391713	0.760631	0.233660	0.047*	
O1C	1.0019 (3)	0.1093 (3)	0.6226 (2)	0.0377 (6)	
H1C	0.985 (5)	0.104 (4)	0.681 (3)	0.057*	
O2C	0.8283 (3)	0.2202 (3)	0.64327 (18)	0.0373 (6)	
N1C	0.8615 (4)	0.2696 (3)	0.4769 (2)	0.0352 (7)	
H11C	0.867623	0.269306	0.415860	0.053*	
H12C	0.923265	0.353762	0.517012	0.053*	
H13C	0.750789	0.243678	0.485806	0.053*	
C1C	0.9138 (4)	0.1728 (3)	0.5965 (2)	0.0268 (6)	
C2C	0.9311 (4)	0.1753 (4)	0.4965 (2)	0.0304 (7)	
H21C	1.052603	0.204456	0.488863	0.037*	
H22C	0.867896	0.084242	0.451120	0.037*	

### Hexaglycinium tetra-µ-iodido-octaiodidotriplumbate Atomic displacement parameters (Å^2^)

**Table:**

	*U*^11^	*U*^22^	*U*^33^	*U*^12^	*U*^13^	*U*^23^
Pb1	0.01827 (7)	0.02247 (7)	0.01990 (7)	0.00920 (6)	0.00231 (5)	0.00423 (6)
Pb2	0.02567 (6)	0.02343 (6)	0.02006 (5)	0.01115 (4)	0.00307 (4)	0.00437 (4)
I1	0.02081 (9)	0.02978 (10)	0.02544 (9)	0.01089 (8)	−0.00022 (7)	0.00788 (8)
I2	0.02197 (9)	0.02590 (10)	0.02631 (9)	0.01158 (7)	0.00746 (7)	0.00847 (7)
I3	0.02360 (9)	0.02525 (10)	0.02430 (9)	0.00811 (7)	0.00517 (7)	0.00853 (7)
I4	0.04911 (14)	0.02858 (11)	0.02416 (10)	0.00690 (10)	0.00171 (9)	0.01154 (8)
I5	0.02567 (10)	0.03563 (12)	0.03222 (11)	0.00594 (9)	0.00550 (8)	0.01039 (9)
I6	0.04793 (14)	0.03453 (12)	0.04935 (14)	0.02767 (11)	0.02635 (11)	0.02394 (11)
O1A	0.0489 (16)	0.0251 (12)	0.0306 (13)	0.0009 (11)	−0.0040 (11)	0.0096 (10)
O2A	0.0541 (16)	0.0256 (12)	0.0280 (12)	0.0052 (11)	−0.0012 (11)	0.0104 (10)
C1A	0.0240 (14)	0.0240 (15)	0.0280 (15)	0.0063 (12)	0.0050 (12)	0.0083 (12)
C2A	0.0415 (19)	0.0234 (16)	0.0288 (16)	0.0057 (14)	0.0033 (14)	0.0099 (13)
N1A	0.0476 (18)	0.0240 (14)	0.0322 (15)	0.0063 (13)	0.0139 (13)	0.0092 (12)
O1B	0.0322 (13)	0.0411 (15)	0.0307 (12)	0.0164 (11)	0.0089 (10)	0.0055 (11)
O2B	0.0351 (13)	0.0478 (15)	0.0253 (11)	0.0217 (12)	0.0083 (10)	0.0096 (11)
C1B	0.0346 (17)	0.0278 (16)	0.0269 (15)	0.0170 (14)	0.0112 (13)	0.0137 (13)
C2B	0.0414 (19)	0.0333 (18)	0.0260 (15)	0.0214 (15)	0.0051 (13)	0.0093 (13)
N1B	0.0427 (17)	0.0304 (15)	0.0267 (13)	0.0176 (13)	0.0148 (12)	0.0107 (12)
O1C	0.0382 (14)	0.0470 (16)	0.0442 (15)	0.0249 (12)	0.0123 (11)	0.0273 (13)
O2C	0.0384 (14)	0.0449 (15)	0.0391 (14)	0.0247 (12)	0.0158 (11)	0.0166 (12)
N1C	0.0302 (15)	0.0430 (17)	0.0437 (17)	0.0180 (13)	0.0108 (13)	0.0254 (14)
C1C	0.0216 (14)	0.0259 (15)	0.0329 (16)	0.0064 (12)	0.0034 (12)	0.0134 (13)
C2C	0.0298 (16)	0.0336 (18)	0.0345 (17)	0.0163 (14)	0.0081 (13)	0.0151 (14)

### Hexaglycinium tetra-µ-iodido-octaiodidotriplumbate Geometric parameters (Å, º)

**Table:**

Pb1—I1^i^	3.1575 (3)	O1B—C1B	1.302 (4)
Pb1—I1	3.1575 (2)	O1B—H1B	0.82 (5)
Pb1—I2^i^	3.1988 (2)	O2B—C1B	1.210 (4)
Pb1—I2	3.1988 (2)	C1B—C2B	1.499 (4)
Pb1—I3	3.2432 (3)	C2B—N1B	1.476 (4)
Pb1—I3^i^	3.2432 (3)	C2B—H21B	0.9900
Pb2—I4	3.0213 (3)	C2B—H22B	0.9900
Pb2—I6	3.0663 (3)	N1B—H11B	0.9100
Pb2—I5	3.0926 (3)	N1B—H12B	0.9100
Pb2—I1	3.4049 (3)	N1B—H13B	0.9100
Pb2—I2	3.4063 (3)	O1C—C1C	1.322 (4)
O1A—C1A	1.304 (4)	O1C—H1C	0.90 (5)
O1A—H1A	0.79 (5)	O2C—C1C	1.196 (4)
O2A—C1A	1.209 (4)	N1C—C2C	1.477 (4)
C1A—C2A	1.502 (4)	N1C—H11C	0.9100
C2A—N1A	1.474 (4)	N1C—H12C	0.9100
C2A—H21A	0.9900	N1C—H13C	0.9100
C2A—H22A	0.9900	C1C—C2C	1.507 (5)
N1A—H11A	0.9100	C2C—H21C	0.9900
N1A—H12A	0.9100	C2C—H22C	0.9900
N1A—H13A	0.9100		
			
I1^i^—Pb1—I1	180.0	C2A—N1A—H12A	109.5
I1^i^—Pb1—I2^i^	91.363 (7)	H11A—N1A—H12A	109.5
I1—Pb1—I2^i^	88.637 (8)	C2A—N1A—H13A	109.5
I1^i^—Pb1—I2	88.637 (7)	H11A—N1A—H13A	109.5
I1—Pb1—I2	91.363 (7)	H12A—N1A—H13A	109.5
I2^i^—Pb1—I2	180.0	C1B—O1B—H1B	109.5
I1^i^—Pb1—I3	88.797 (5)	O2B—C1B—O1B	126.6 (3)
I1—Pb1—I3	91.203 (5)	O2B—C1B—C2B	121.3 (3)
I2^i^—Pb1—I3	91.693 (5)	O1B—C1B—C2B	112.1 (3)
I2—Pb1—I3	88.307 (5)	N1B—C2B—C1B	110.1 (3)
I1^i^—Pb1—I3^i^	91.203 (5)	N1B—C2B—H21B	109.6
I1—Pb1—I3^i^	88.797 (6)	C1B—C2B—H21B	109.6
I2^i^—Pb1—I3^i^	88.307 (5)	N1B—C2B—H22B	109.6
I2—Pb1—I3^i^	91.693 (5)	C1B—C2B—H22B	109.6
I3—Pb1—I3^i^	180.0	H21B—C2B—H22B	108.2
I4—Pb2—I6	92.576 (7)	C2B—N1B—H11B	109.5
I4—Pb2—I5	88.198 (8)	C2B—N1B—H12B	109.5
I6—Pb2—I5	98.865 (9)	H11B—N1B—H12B	109.5
I4—Pb2—I1	98.685 (7)	C2B—N1B—H13B	109.5
I6—Pb2—I1	166.133 (7)	H11B—N1B—H13B	109.5
I5—Pb2—I1	89.602 (8)	H12B—N1B—H13B	109.5
I4—Pb2—I2	88.550 (7)	C1C—O1C—H1C	109.5
I6—Pb2—I2	88.468 (8)	C2C—N1C—H11C	109.5
I5—Pb2—I2	172.104 (7)	C2C—N1C—H12C	109.5
I1—Pb2—I2	83.779 (8)	H11C—N1C—H12C	109.5
Pb1—I1—Pb2	76.444 (6)	C2C—N1C—H13C	109.5
Pb1—I2—Pb2	75.892 (6)	H11C—N1C—H13C	109.5
C1A—O1A—H1A	109.5	H12C—N1C—H13C	109.5
O2A—C1A—O1A	125.6 (3)	O2C—C1C—O1C	126.1 (3)
O2A—C1A—C2A	122.0 (3)	O2C—C1C—C2C	123.3 (3)
O1A—C1A—C2A	112.4 (3)	O1C—C1C—C2C	110.6 (3)
N1A—C2A—C1A	109.8 (3)	N1C—C2C—C1C	108.8 (3)
N1A—C2A—H21A	109.7	N1C—C2C—H21C	109.9
C1A—C2A—H21A	109.7	C1C—C2C—H21C	109.9
N1A—C2A—H22A	109.7	N1C—C2C—H22C	109.9
C1A—C2A—H22A	109.7	C1C—C2C—H22C	109.9
H21A—C2A—H22A	108.2	H21C—C2C—H22C	108.3
C2A—N1A—H11A	109.5		

Symmetry code: (i) −*x*+1, −*y*+1, −*z*+1.

### Hexaglycinium tetra-µ-iodido-octaiodidotriplumbate Hydrogen-bond geometry (Å, º)

**Table:**

*D*—H···*A*	*D*—H	H···*A*	*D*···*A*	*D*—H···*A*
O1*A*—H1*A*···O2*A*^ii^	0.79 (5)	1.85 (3)	2.638 (4)	171 (4)
C2*A*—H21*A*···I1^iii^	0.99	3.10	3.669 (3)	118
C2*A*—H22*A*···I2	0.99	3.33	4.271 (4)	160
N1*A*—H11*A*···I4^iv^	0.91	3.05	3.549 (3)	116
N1*A*—H11*A*···I5^iii^	0.91	3.27	3.983 (3)	137
N1*A*—H11*A*···I6	0.91	3.04	3.655 (3)	126
N1*A*—H12*A*···I5^iv^	0.91	2.69	3.588 (3)	170
N1*A*—H13*A*···I4	0.91	2.75	3.605 (3)	156
O1*B*—H1*B*···O2*B*^v^	0.82 (4)	1.85 (4)	2.667 (3)	176 (4)
C2*B*—H21*B*···I2	0.99	3.03	3.976 (3)	161
C2*B*—H22*B*···I6^vi^	0.99	3.18	3.775 (3)	120
N1*B*—H11*B*···I3^i^	0.91	2.82	3.648 (3)	151
N1*B*—H12*B*···I1	0.91	3.11	3.647 (3)	119
N1*B*—H12*B*···O1*A*^vii^	0.91	2.49	3.320 (4)	151
N1*B*—H13*B*···I6^vi^	0.91	2.79	3.528 (3)	139
O1*C*—H1*C*···I5^viii^	0.90 (5)	2.57 (3)	3.445 (3)	164 (4)
N1*C*—H11*C*···I1^iii^	0.91	3.09	3.711 (3)	127
N1*C*—H11*C*···I2	0.91	3.13	3.640 (3)	117
N1*C*—H11*C*···I6	0.91	3.31	3.868 (3)	122
N1*C*—H12*C*···I2^ix^	0.91	3.00	3.779 (3)	145
N1*C*—H13*C*···I3	0.91	2.73	3.628 (3)	170
C2*C*—H21*C*···I3^iii^	0.99	3.14	4.068 (3)	156

Symmetry codes: (i) −*x*+1, −*y*+1, −*z*+1; (ii) −*x*+2, −*y*+1, −*z*; (iii) *x*+1, *y*, *z*; (iv) −*x*+1, −*y*, −*z*; (v) −*x*+1, −*y*+1, −*z*; (vi) *x*, *y*+1, *z*; (vii) *x*−1, *y*, *z*; (viii) −*x*+1, −*y*, −*z*+1; (ix) −*x*+2, −*y*+1, −*z*+1.
