# Supplementary material for: Crystal structure of hexa­glycinium dodeca­iodo­triplumbate
Source: Acta Crystallogr E Crystallogr Commun. 2024 Aug 6;80(Pt 9):916–20. doi: 10.1107/S2056989024007606 (PMC11389678; doi:10.1107/S2056989024007606)
Supplement: Supplementary file 4 [file e-80-00916-sup5.docx]

**Supporting information**

**Supplementary crystallographic information**

**Supporting crystallographic data**

| C_12_H_36_I_12_N_6_O_12_Pb_3_ | *F*(000) = 1128 |
| --- | --- |
| *M_r_* = 2600.84 | *D*_x_ = 3.421 Mg m^−3^ |
| *a* = 8.5437(5) Å | Mo *K*α radiation, λ = 0.71073 Å |
| *b* = 11.2672(7) Å | Cell parameters from 9918 reflections |
| *c* = 14.8534(9) Å | θ = 2.045–33.210° |
| *α* = 105.900(2)° | µ = 17.36 mm^−1^ |
| *β* = 92.647(2)° | *T* = 200 K |
| *γ* = 111.477(2)° | Plate, yellow |
| *V* = 1262.40(13) Å^3^ | 0.1 × 0.08 × 0.06 mm^3^ |
| *Z* = 1 |  |

**Data collection**

| Bruker APEXII diffractometer equipped with a CCD area detector | 8389 reflections with *I* > 2σ(*I*) |
| --- | --- |
| φ and ω scans | *R*_int_ = 0.0293 |
| Absorption correction: multi-scan (SADABS; Krause *et al.*, 2015) | θ_max_ = 33.210°, θ_min_ = 2.045° |
| *T*_min_ = 0.548, *T*_max_ = 0.747 absorpt_correction_ | *h* = −13→13 |
| 54402 measured reflections | *k* = −17→17 |
| 9640 independent reflections | *l* = −22→22 |

Krause, L., Herbst-Irmer, R., Sheldrick, G. M. & Stalke, D. (2015). *J. Appl. Cryst.* **48**, 3–10.

**Refinement**

| Refinement on *F*^2^ | 0 restraints |
| --- | --- |
| Least-squares matrix: full | Hydrogen site location: mixed |
| *R*[*F*^2^ > 2σ(*F*^2^)] = 0.0216 | H atoms treated by constrained refinement |
| *wR*(*F*^2^) = 0.0385 | *w* = 1/[σ^2^(*F*_o_^2^) + (0.09*P*)^2^ + 2.57*P*] where *P* = (*F*_o_^2^ + 2*F*_c_^2^)/3 |
| *S* = 1.028 | (Δ/σ)_max_ = 0.002 |
| 8389 reflections with *F*^2^ > 2σ(*F*^2^) | Δρ_max_ = 2.294 e Å^−3^ |
| 215 parameters | Δρ_min_ = −2.485 e Å^−3^ |

**Special details**

**Geometry.** All esds (except the esd in the dihedral angle between two l.s. planes) are estimated using the full covariance matrix. The cell esds are taken into account individually in the estimation of esds in distances, angles and torsion angles; correlations between esds in cell parameters are only used when they are defined by crystal symmetry. An approximate (isotropic) treatment of cell esds is used for estimating esds involving l.s. planes.

**Fractional atomic coordinates and isotropic or equivalent isotropic displacement parameters (Å^2^)**

|  | *x* | *y* | *z* | *U*_iso_*/*U*_eq_ |
| --- | --- | --- | --- | --- |
| Pb1 | 0.500000 | 0.500000 | 0.500000 | 0.02041(3) |
| Pb2 | 0.38342(2) | 0.18885(2) | 0.25654(2) | 0.02316(3) |
| I1 | 0.19064(2) | 0.40522(2) | 0.33606(2) | 0.02537(4) |
| I2 | 0.74935(2) | 0.46827(2) | 0.34936(2) | 0.02390(4) |
| I3 | 0.41471(2) | 0.19237(2) | 0.49364(2) | 0.02463(4) |
| I4 | 0.42551(3) | 0.22381(2) | 0.06337(2) | 0.03591(5) |
| I5 | 0.03007(3) | -0.03864(2) | 0.16590(2) | 0.03305(5) |
| I6 | 0.59810(3) | 0.01929(2) | 0.23522(2) | 0.03749(6) |
| O1A | 1.0289(4) | 0.4863(2) | 0.11937(18) | 0.0392(6) |
| H1A | 1.044(5) | 0.540(3) | 0.0923(19) | 0.059* |
| O2A | 0.8915(4) | 0.3408(2) | -0.02294(17) | 0.0389(6) |
| C1A | 0.9400(4) | 0.3667(3) | 0.0605(2) | 0.0263(6) |
| C2A | 0.9034(5) | 0.2593(3) | 0.1076(2) | 0.0330(7) |
| H21A | 1.011081 | 0.252552 | 0.127759 | 0.040* |
| H22A H | 0.852011 | 0.282924 | 0.164719 | 0.040* |
| N1A | 0.7854(4) | 0.1293(3) | 0.0409(2) | 0.0366(7) |
| H11A | 0.774867 | 0.062872 | 0.066806 | 0.055* |
| H12A H | 0.826984 | 0.112846 | -0.014398 | 0.055* |
| H13A H | 0.681464 | 0.132077 | 0.029277 | 0.055* |
| O1B | 0.6686(3) | 0.5891(3) | 0.10354(18) | 0.0352(6) |
| H1B | 0.6483(17) | 0.542(5) | 0.048(3) | 0.053* |
| O2B | 0.3916(3) | 0.5535(3) | 0.07956(16) | 0.0347(5) |
| C1B | 0.5289(4) | 0.5982(3) | 0.1296(2) | 0.0270(6) |
| C2B | 0.5538(5) | 0.6726(4) | 0.2331(2) | 0.0316(7) |
| H21B | 0.578315 | 0.619973 | 0.271200 | 0.038* |
| H22B | 0.652301 | 0.759908 | 0.248893 | 0.038* |
| N1B | 0.3990(4) | 0.6954(3) | 0.2566(2) | 0.0316(6) |
| H11B | 0.404495 | 0.721763 | 0.320722 | 0.047* |
| H12B | 0.305401 | 0.618023 | 0.229916 | 0.047* |
| H13B | 0.391713 | 0.760631 | 0.233660 | 0.047* |
| O1C | 1.0019(3) | 0.1093(3) | 0.6226(2) | 0.0377(6) |
| H1C | 0.985(5) | 0.104(4) | 0.681(3) | 0.057* |
| O2C | 0.8283(3) | 0.2202(3) | 0.64327(18) | 0.0373(6) |
| N1C | 0.8615(4) | 0.2696(3) | 0.4769(2) | 0.0352(7) |
| H11C | 0.867623 | 0.269306 | 0.415860 | 0.053* |
| H12C | 0.923265 | 0.353762 | 0.517012 | 0.053* |
| H13C | 0.750789 | 0.243678 | 0.485806 | 0.053* |
| C1C | 0.9138(4) | 0.1728(3) | 0.5965(2) | 0.0268(6) |
| C2C | 0.9311(4) | 0.1753(4) | 0.4965(2) | 0.0304(7) |
| H21C | 1.052603 | 0.204456 | 0.488863 | 0.037* |
| H22C | 0.867896 | 0.084242 | 0.451120 | 0.037* |

**Atomic displacement parameters (Å^2^)**

|  | *U*^11^ | *U*^22^ | *U*^33^ | *U*^12^ | *U*^13^ | *U*^23^ |
| --- | --- | --- | --- | --- | --- | --- |
| Pb1 | 0.01827(7) | 0.02247(7) | 0.01990(7) | 0.00920(6) | 0.00231(5) | 0.00423(6) |
| Pb2 | 0.02567(6) | 0.02343(6) | 0.02006(5) | 0.01115(4) | 0.00307(4) | 0.00437(4) |
| I1 | 0.02081(9) | 0.02978(10) | 0.02544(9) | 0.01089(8) | -0.00022(7) | 0.00788(8) |
| I2 | 0.02197(9) | 0.02590(10) | 0.02631(9) | 0.01158(7) | 0.00746(7) | 0.00847(7) |
| I3 | 0.02360(9) | 0.02525(10) | 0.02430(9) | 0.00811(7) | 0.00517(7) | 0.00853(7) |
| I4 | 0.04911(14) | 0.02858(11) | 0.02416(10) | 0.00690(10) | 0.00171(9) | 0.01154(8) |
| I5 | 0.02567(10) | 0.03563(12) | 0.03222(11) | 0.00594(9) | 0.00550(8) | 0.01039(9) |
| I6 | 0.04793(14) | 0.03453(12) | 0.04935(14) | 0.02767(11) | 0.02635(11) | 0.02394(11) |
| O1A | 0.0489(16) | 0.0251(12) | 0.0306(13) | 0.0009(11) | -0.0040(11) | 0.0096(10) |
| O2A | 0.0541(16) | 0.0256(12) | 0.0280(12) | 0.0052(11) | -0.0012(11) | 0.0104(10) |
| C1A | 0.0240(14) | 0.0240(15) | 0.0280(15) | 0.0063(12) | 0.0050(12) | 0.0083(12) |
| C2A | 0.0415(19) | 0.0234(16) | 0.0288(16) | 0.0057(14) | 0.0033(14) | 0.0099(13) |
| N1A | 0.0476(18) | 0.0240(14) | 0.0322(15) | 0.0063(13) | 0.0139(13) | 0.0092(12) |
| O1B | 0.0322(13) | 0.0411(15) | 0.0307(12) | 0.0164(11) | 0.0089(10) | 0.0055(11) |
| O2B | 0.0351(13) | 0.0478(15) | 0.0253(11) | 0.0217(12) | 0.0083(10) | 0.0096(11) |
| C1B | 0.0346(17) | 0.0278(16) | 0.0269(15) | 0.0170(14) | 0.0112(13) | 0.0137(13) |
| C2B | 0.0414(19) | 0.0333(18) | 0.0260(15) | 0.0214(15) | 0.0051(13) | 0.0093(13) |
| N1B | 0.0427(17) | 0.0304(15) | 0.0267(13) | 0.0148(12) | 0.0148(12) | 0.0107(12) |
| O1C | 0.0382(14) | 0.0470(16) | 0.0442(15) | 0.0249(12) | 0.0123(11) | 0.0273(13) |
| O2C | 0.0384(14) | 0.0449(15) | 0.0391(14) | 0.0247(12) | 0.0158(11) | 0.0166(12) |
| N1C | 0.0302(15) | 0.0430(17) | 0.0437(17) | 0.0180(13) | 0.0108(13) | 0.0254(14) |
| C1C | 0.0216(14) | 0.0259(15) | 0.0329(16) | 0.0064(12) | 0.0034(12) | 0.0134(13) |
| C2C | 0.0298(16) | 0.0336(18) | 0.0345(17) | 0.0163(14) | 0.0081(13) | 0.0151(14) |

**Geometric parameters (Å,°)**

| C1A-O1A | 1.304(4) | C1B-O1B | 1.302(4) | C1C-O1C | 1.322(4) |
| --- | --- | --- | --- | --- | --- |
| C1A-O2A | 1.209(4) | C1B-O2B | 1.210(4) | C1C-O2C | 1.196(4) |
| C1A-C2A | 1.502(4) | C1B-C2B | 1.499(4) | C1C-C2C | 1.507(5) |
| O1A-H1A | 0.79(5) | O1B-H1B | 0.82(5) | O1C-H1C | 0.90(5) |
| C2A-N1A | 1.474(4) | C2B-N1B | 1.476(4) | C2C-N1C | 1.477(4) |
| C2A-H21A | 0.9900 | C2B-H21B | 0.9900 | C2C-H21C | 0.9900 |
| C2A-H22A | 0.9900 | C2B-H22B | 0.9900 | C2C-H22C | 0.9900 |
| N1A-H11A | 0.9100 | N1B-H11B | 0.9100 | N1C-H11C | 0.9100 |
| N1A-H12A | 0.9100 | N1B-H12B | 0.9100 | N1C-H12C | 0.9100 |
| N1A-H13A | 0.9100 | N1B-H13B | 0.9100 | N1C-H13C | 0.9100 |
| Pb1-I1 | 3.1575(3) 2× | Pb2-I1 | 3.4049(3) | Pb2-I4 | 3.0213(3) |
| Pb1-I2 | 3.1988(2) 2× | Pb2-I2 | 3.4063(3) | Pb2-I5 | 3.0926(3) |
| Pb1-I3 | 3.2432(3) 2× | I2-Pb1-I3 | 88.307(5) | Pb2-I6 | 3.0663(3) |
| I1-Pb1-I1 | 180.0 | I2-Pb1-I3 | 91.693(5) | Pb1 I1 Pb2 | 76.444(6) |
| I1-Pb1-I2 | 91.363(7) | I3-Pb1-I3 | 180.0 | Pb1 I2 Pb2 | 75.892(6) |
| I1-Pb1-I2 | 88.637(8) | I4-Pb2-I6 | 92.576(7) | C1A-O1A-H1A | 109.5 |
| I1-Pb1-I2 | 88.637(7) | I4-Pb2-I5 | 88.198(8) | O2A-C1A-O1A | 125.6(3) |
| I1-Pb1-I2 | 91.363(7) | I6-Pb2-I5 | 98.865(9) | O2A-C1A-C2A | 122.0(3) |
| I2-Pb1-I2 | 180.0 | I4-Pb2-I1 | 98.685(7) | O1A-C1A-C2A | 112.4(3) |
| I1-Pb1-I3 | 88.797(5) | I6-Pb2-I1 | 166.133(7) | N1A-C2A-C1A | 109.8(3) |
| I1-Pb1-I3 | 91.203(5) | I5-Pb2-I1 | 89.602(8) | N1A-C2A-H21A | 109.7 |
| I2-Pb1-I3 | 91.693(5) | I4-Pb2-I2 | 88.550(7) | C1A-C2A-H21A | 109.7 |
| I2-Pb1-I3 | 88.307(5) | I6-Pb2-I2 | 88.468(8) | N1A-C2A-H22A | 109.7 |
| I1-Pb1-I3 | 91.203(5) | I5-Pb2-I2 | 172.104(7) | C1A-C2A-H22A | 109.7 |
| I1-Pb1-I3 | 88.797(6) | I1-Pb2-I2 | 83.779(8) | H21A-C2A-H22A | 108.2 |
| C2A-N1A-H11A | 109.5 | H11A-N1A-H13A | 109.5 | O2B-C1B-C2B | 121.3(3) |
| C2A-N1A-H12A | 109.5 | H12A-N1A-H13A | 109.5 | O1B-C1B-C2B | 112.1(3) |
| H11A-N1A-H12A | 109.5 | C1B-O1B-H1B | 109.5 | N1B-C2B-C1B | 110.1(3) |
| C2A-N1A-H13A | 109.5 | O2B-C1B-O1B | 126.6(3) | N1B-C2B-H21B | 109.6 |
| C1B-C2B-H21B | 109.6 | H12B-N1B-H13B | 109.5 | C1CC2C-H21C | 109.9 |
| N1B-C2B-H22B | 109.6 | C1C-O1C-H1C | 109.5 | N1C-C2C-H22C | 109.9 |
| C1B-C2B-H22B | 109.6 | O2C-C1C-C2C | 123.3(3) | C1C-C2C-H22C | 109.9 |
| H21B-C2B-H22B | 108.2 | C2C-N1C-H11C | 109.5 | H21C-C2C-H22C | 108.3 |
| C2B-N1B-H11B | 109.5 | C2C-N1C-H12C | 109.5 | O1C-C1C-C2C | 110.6(3) |
| C2B-N1B-H12B | 109.5 | H11C-N1C-H12C | 109.5 | N1C-C2C-C1C | 108.8(3) |
| H11B-N1B-H12B | 109.5 | C2C-N1C-H13C | 109.5 | N1C-C2C-H21C | 109.9 |
| C2B-N1B-H13B | 109.5 | H11C-N1C-H13C | 109.5 | O2C-C1C-O1C | 126.1(3) |
| H11B-N1B-H13B | 109.5 | H12C-N1C-H13C | 109.5 |  |  |

**Hydrogen bind geometry (Å,°)**

| D-H···A | D-H | H···A | D···A | ∠DHA |
| --- | --- | --- | --- | --- |
| O1A-H1A···O2A ^i^ | 0.79 | 1.85 | 2.637(3) | 171 |
| O1B-H1B···O2B ^ii^ | 0.82 | 1.85 | 2.667(3) | 175 |
| O1C-H1C···I5 ^iii^ | 0.90 | 2.57 | 3.445(3) | 164 |
| N1A-H11A···I4 ^iv^ | 0.91 | 3.05 | 3.549(3) | 116 |
| N1A-H11A···I6 | 0.91 | 3.04 | 3.655(3) | 126 |
| N1A-H12A···I5 ^iv^ | 0.91 | 2.69 | 3.588(3) | 170 |
| N1A-H13A···I4 | 0.91 | 2.75 | 3.605(3) | 156 |
| C2A-H21A···I1 ^v^ | 0.99 | 3.10 | 3.669(3) | 118 |
| N1B-H11B···I3 ^vi^ | 0.91 | 2.82 | 3.648(3) | 175 |
| N1B-H12B···I1 | 0.91 | 3.11 | 3.647(3) | 119 |
| N1B-H13B···I6 ^vii^ | 0.91 | 2.79 | 3.528(3) | 139 |
| C2B-H21B···I2 | 0.99 | 3.03 | 3.976(3) | 161 |
| N1C-H11C···I1 ^v^ | 0.91 | 3.09 | 3.711(3) | 127 |
| N1C-H11C···I2 | 0.91 | 3.13 | 3.640(3) | 117 |
| N1C-H12C···I2 ^viii^ | 0.91 | 3.00 | 3.779(3) | 145 |
| N1C-H13C···I3 | 0.91 | 2.73 | 3.628(3) | 170 |
| C2C-H21C···I3 ^v^ | 0.99 | 3.14 | 4.068(3) | 156 |

Symmetry codes: (i) –x+2, -y+1, -z; (ii) –x+1, -y+1, -z; (iii) –x+1, -y, -z+1; (iv) –x+1, -y, -z; (v) x+1, y, z; (vi) –x+1, -y+1, -z+1; (vii) x, y+1, z; (viii) –x+2, -y+1, -z+1.
